# Psychomotor Behavior: A Practical Approach in *Drosophila*

**DOI:** 10.3389/fpsyt.2016.00153

**Published:** 2016-08-31

**Authors:** Konstantin G. Iliadi, Oxana B. Gluscencova, Gabrielle L. Boulianne

**Affiliations:** ^1^Program in Developmental and Stem Cell Biology, The Hospital For Sick Children, Toronto, ON, Canada; ^2^Department of Molecular Genetics, University of Toronto, Toronto, ON, Canada

**Keywords:** *Drosophila*, locomotion, psychomotor learning, neurodegeneration, behavior

## Abstract

Psychomotor behaviors are governed by fine relationships between physical activity and cognitive functions. Disturbances in psychomotor development and performance are a hallmark of many mental illnesses and often appear as observable and measurable behaviors. Here, we describe a new method called an “equilibrist test,” which can be used to quantify psychomotor learning and performance in *Drosophila*. We also show how this test can be used to quantify motor disturbances at relatively early stages in the development of neurodegenerative diseases.

## Introduction

Many psychiatric disorders are thought to arise as a result of cumulative effects of multiple risk factors (1, 2). This complex pattern of heritability together with the heterogeneous nature of human mental illness has made it difficult to identify specific causes of disease. Recent studies, however, have shown that at least some mental illnesses may result from mutations in single genes including Disrupted in Schizophrenia-1 (DISC-1), which was first identified in a large Scottish family with high rates of schizophrenia, bipolar disorder, and depression (3). Single gene mutations have also been associated with numerous other disorders including Fragile X syndrome (FMR1) (4), Rett syndrome (MECP2), and autism spectrum disorder (NLGN 3 and 4 – neuroligin) (5). Moreover, the development of high-throughput DNA sequencing technologies has led to the identification of numerous mutations that are linked to various neurodevelopmental and neuropsychiatric disorders. Interestingly, many of these genes are conserved in *Drosophila*. Indeed, previous studies have shown that 714 distinct human disease genes match 548 unique *Drosophila* sequences (6). Moreover, some of the genes that have been implicated in the pathogenesis of psychiatric diseases have counterparts in the *Drosophila* genome (7, 8). Taken together, these findings suggest that there may be an opportunity to study neuropsychiatric disorders in fruit flies. However, whether psychomotor activity can be measured in *Drosophila* remains unclear.

In humans, the signs and symptoms of mental illness can be highly variable and appear as patterns of abnormal thoughts and emotions, as well as maladaptive behavior, and social interaction. Among these symptoms, psychomotor disturbances are common in cases of severe depression (9), bipolar disorder (10), and schizophrenia (11). Psychomotor activity can be defined as the fine relationships between several general symptom categories such as sensory perception, cognition, emotion, and movement (12, 13). Abnormalities in psychomotor activity can manifest in different domains including gross and fine motor activity and body movements, as well as speech and motor response time (14).

The objectives of this study were to develop a novel assay to measure psychomotor activity and learning in *Drosophila*. For this purpose, we used flies carrying a mutation in the *iPLA2-VIA* gene, the *Drosophila* homolog of human PLA2G6. *PLA2G6* encodes a calcium-independent phospholipase A2 enzyme that catalyzes the hydrolysis of glycerophospholipids. In humans, mutations in *PLA2G6* lead to a spectrum of hereditary disorders classified as neurodegeneration with brain iron accumulation (NBIA) (15), infantile neuroaxonal dystrophy (INAD) (16), adult dystonia-parkinsonism (17), and Parkinson disease (18). *PLA2G6*-associated disorders appear as a wide range of overlapping phenotypes such as progressive psychomotor delay and impairments in psychomotor learning, gait instability and disturbance, rapid cognitive decline, and neuropsychiatric changes.

## Materials and Methods

### Fly Stocks and Maintenance

All stocks were raised on standard fly food, with a 12/12-h light/dark cycle, at 24 ± 1°C and 45–50% relative humidity. The *Canton S* (*CS*) line was used as the wild type control. The P-element insertion mutant line *y*^1^, *w*^67c23^; EPgy2-iPLA2-VIA^EY05103^ was obtained from the *Drosophila* Bloomington Stock Center (stock number 15947). The P-element is inserted in the 5′ untranslated region (UTR) of the *iPLA2-VIA* gene (CG6718), which encodes a calcium-independent phospholipase A2-VIA. To equilibrate the genetic background, the P-element mutant line was backcrossed five times to a wild type *CS* control line. Experimental flies were raised and maintained under non-crowded conditions.

### Video Tracking and Data Analysis

All behavioral experiments were performed with wing-clipped males within a 3-h time window (between 16.00 and 19.00 hours) in an environmental control room. The wings of males were clipped at least 3 days before experiments were performed under light CO_2_ anesthesia.

To test spontaneous locomotor activity, we used modified six-well tissue culture plates (Falcon#353046, 35 mm diameter and 7 mm high). Individual flies were gently aspirated into the circular chamber 30 min prior to recording activity to allow them to adapt to the new environment. A color camera (EverFocus Eq. 610, Polistar II) was fitted with a CCTV lens (Computar, Vari Focal TG4Z2813 FCS-IR) and fixed on a mounting bracket about 50 cm above the chambers. The distance of the camera to the object as well as the zoom, focus, and iris aperture were optimized for video tracking. The path of freely moving flies within the arena was tracked during 10 min with Ethovioson XT v.10 (Noldus Information Technology, Leesburg, VA, USA).

The setup for testing psychomotor activity and learning consists of two platforms submerged within a pool, with only the small upper parts 5 mm over the water surface. A clear fishing line (0.6 mm) was then strung between two platforms. A single fly was then gently removed from a vial by aspirator to the thorax and placed on the platform. Video tracking was initiated when the fly entered a zone (colored in red), which is 1 cm away from both platforms and recordings continued until the fly traveled the total distance (13 cm) or when 60 s had elapsed. The platform was made from polyethylene disconnector (Part No: 1136651 Kartell labware) that was glued to the bottom of the pool. The learning protocol consisted of three sessions, each separated by 1 day. Each session consists of three successive trials (without intervals in between).

The trajectories obtained from video tracking were analyzed for the following parameters: mean of total distance covered in 10 min, mean of percent of time spent in total activity, mean of walking speed during locomotor activity, and mean of walking speed during the “equilibrist test.” To reduce any effects due to non-specific jerky movements and/or wobbling, we defined a fly to be moving when it had traveled a minimum of 4 mm/s and a fly to be stopped when its speed was 2 mm/s or less during locomotor activity. For the “equilibrist test,” we defined a fly to be moving when its speed was 2 mm/s or higher. These parameters were averaged for five data points as a sliding window over the total recording time (19). For all behavioral assays, a double-blind method was employed.

### Morphometrics

The width of a male thorax was measured as the distance between the posterior sternopleural bristles on the ventral surface of the thorax (20). Fly images were captured using the DEM200 digital camera attached to a Nikon SMZ645 stereoscope and then analyzed with micro-measure 1.20 software.

### Statistical Procedures

The morphological data (thorax width) were tested for homogeneity (Levene-test) and then compared by unpaired *t*-test. For locomotor activity, boxes show the median and upper and lower quartiles (25–75%); whiskers extend from the minimum to the maximum. The data were compared using the Mann–Whitney *U* test. A general linear model (GLM) was constructed to estimate the association between performance in “equilibrist test” (mean of walking speed) and number of trials. *Post hoc* analysis was conducted using Tukey HSD method.

## Results

In humans, psychomotor activity can be measured by clinical assessments or questionnaires (12). In animal models, particularly mice and rats, a wide variety of different behavioral tests can be used to examine psychomotor performance including, but not limited to, an open field test, elevated plus maze test, accelerated rotarod test, and a forced swimming test. Among these, the open field test is the only one that has previously been validated in *Drosophila*. Another test that is frequently used to study locomotor activity in the fly is a negative geotaxis assay (21–23). A fly’s performance in this assay represents the integration of many activities including negative geotaxis response, climbing ability, escape response, and locomotor activity itself. The assay thereby provides a general method to detect any gross defects in motor activity rather than fine-tune movements, motor coordination, or psychomotor behavior. Gap climbing behavior (24) could possibly be used to study psychomotor behavior in the fly. Under normal circumstances, a fruit fly, with a body size of about 2.5 mm, is able to cross a gap of more than 4.0 mm. Studies have shown that flies first visually measure gap width, and if the gap has a traversable width, they will try to cross. Interestingly, if the gap is short enough, flies engage in an extraordinary crossing behavior that involves a distinct motor program. First, a fly will try to grab the opposing side with its front legs, forming a “bridge” while the hind and middle legs remain on the starting side of the gap. Then the fly will move its hind legs to the border of the gap, the mid legs stretch, lifting up the abdomen, and the front legs finally stretch as much as possible to grasp the other side. Several mutant lines have been identified that display defects in gap climbing. Some of these exhibit specific defects deciding whether to cross (decision-making mutant) while others displayed specific impairments while climbing. Unfortunately, whether flies can improve their performance in gap-crossing behavior after multiple repeated attempts remains unknown.

**Figure 1:**
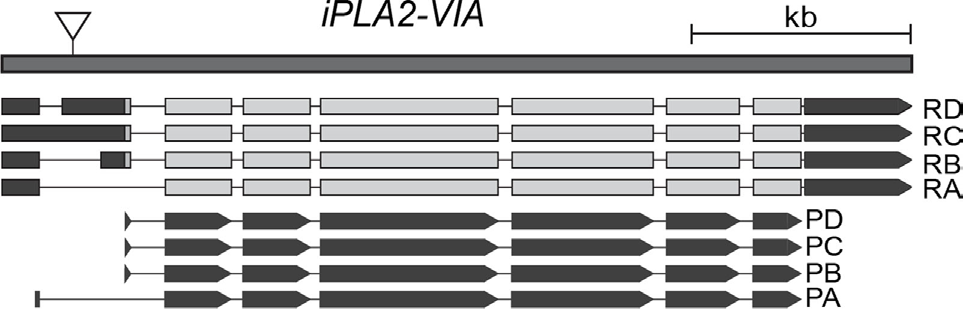
**Schematic representation of the *Drosophila iPLA2-VIA* gene (modified from flybase), showing four annotated transcripts: RA, RB, RC, and RD that give rise to the four protein isoforms PA, PB, PC, and PD**. The position of the P-element insertion is shown as downward-pointing triangle.

To expand the repertoire of assays that can be used to monitor psychomotor activity and learning in *Drosophila*, we used flies carrying a mutation in the *iPLA2-VIA* gene, which encodes the homolog of human PLA2G6. The mutation is caused by the insertion of a transposable P-element in the 5′ UTR of the gene (Figure 1), which results in a severe reduction in expression. The mutant flies also have a reduced lifespan and display widespread vacuolation within the brain as well as mitochondrial degeneration (25).

**Figure 2:**
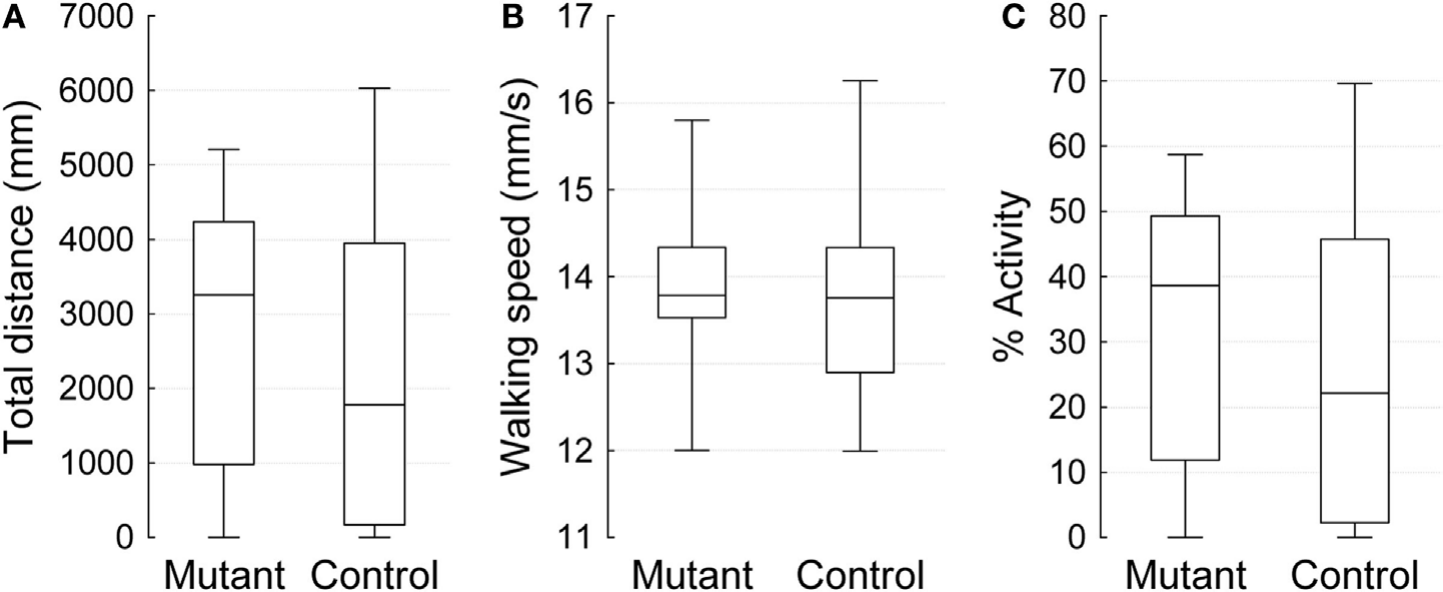
**Open field 10 min video-tracking data for 2-week-old *IPLA2-VIA* mutant and control males**. Plots show the median (horizontal line), 25 and 75% quartiles (box), and extreme (min/max) values (whiskers). **(A)** The total distance covered in 10 min (mm), **(B)** Mean of walking speed (mm/s), and **(C)** Percent activity over time.

**Figure 3:**
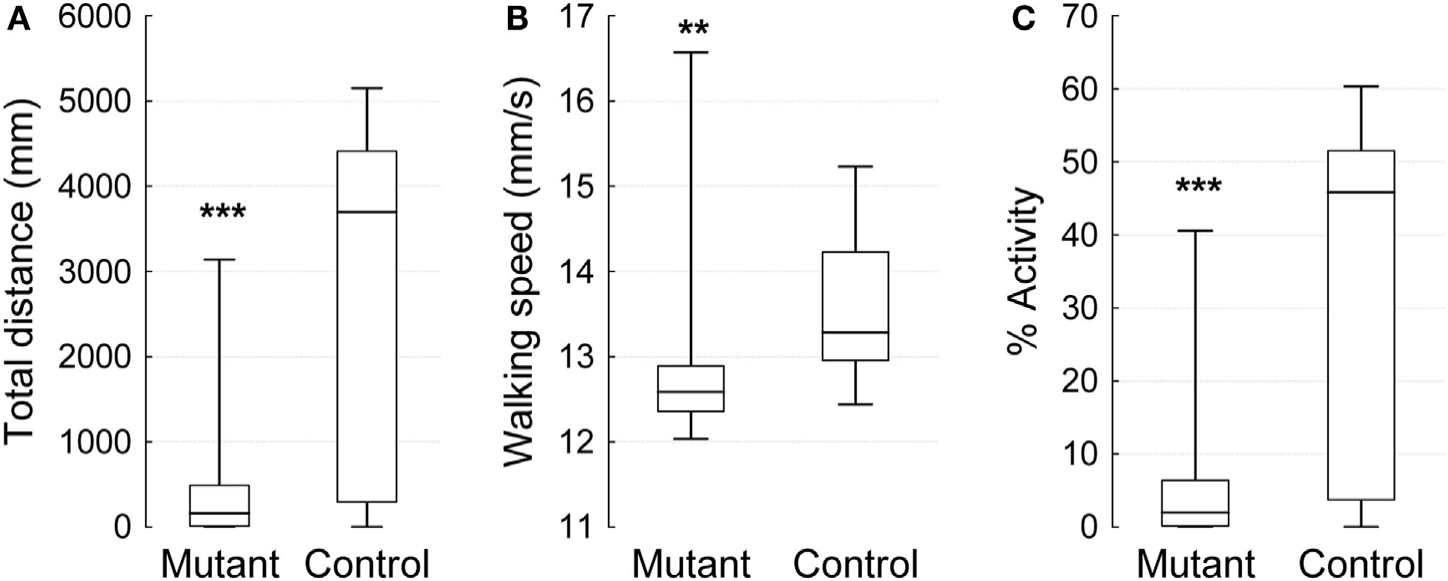
**Open field 10 min video-tracking data for 4-week-old *IPLA2-VIA* mutant and control males**. Plot show the median (horizontal line), 25 and 75% quartiles (box), and extreme (min/max) values (whiskers). **(A)** The total distance covered in 10 min (mm), **(B)** Mean of walking speed (mm/s), and **(C)** Percent of activity over time. Mann–Whitney *U* test (**p < 0.01, ***p < 0.001).

To assess the effect of a mutation in the *Drosophila* homolog of the human PLA2G6 gene on behavior, we first made use of video-assisted motion tracking to quantitate locomotor activity in an open field test. We did not observe any differences between 2-week-old control and mutant flies in the total distance covered (Table 1). Similarly, we did not detect any differences in walking speed or in the percent of time spent in activity (Figure 2). In contrast, 4-week-old mutant flies showed a highly significant, age-dependent reduction for all locomotor parameters: total distance covered, walking speed, and percent of time spent in activity (Table 1 and Figure 3). Although all of the locomotor parameters of 4-week-old mutant flies were significantly reduced compared with control flies, the difference in walking speed (13.28 mm/s for controls vs. 12.36 mm/s for mutants) was not as dramatic compared to the differences we observed between controls and mutants in the total distance covered and the percent of time spent in the activity, which differed by more than fivefold. This would suggest that, while old mutant flies were less active overall, their physical ability to walk in the open field was only slightly affected.

**Table 1:** **Behavioral parameters measured in the open field tests for 2- and 4-week-old *Canton S* control and *IPLA2-VIA* mutant males**.

	*Canton S*	*IPLA2-VIA*	Mann–Whitney *U*
**2-week-old flies**			
Total distance covered (mm)			
Mean ± SE	2128.00 ± 253.32	2671.78 ± 224.11	
Median	1782.35 (*N* = 62)	3253.69 (*N* = 63)	*U* = 1702; *p* = 0.22
Walking speed (mm/s)			
Mean ± SE	13.66 ± 0.13	13.89 ± 0.09	
Median	13.75 (*N* = 56)	13.78 (*N* = 54)	*U* = 1297; *p* = 0.20
Percent of time spent in activity (%)			
Mean ± SE	24.89 ± 2.9	31.81 ± 2.65	
Median	22.13 (*N* = 62)	38.62 (*N* = 63)	*U* = 1684; *p* = 0.18
**4-week-old flies**			
Total distance covered (mm)			
Mean ± SE	2599.13 ± 405.84	480.23 ± 153.25	
Median	3699.91 (*N* = 26)	163.22 (*N* = 26)	*U* = 141; *p* < 0.001
Walking speed (mm/s)			
Mean ± SE	13.59 ± 0.17	13.05 ± 0.28	
Median	13.28 (*N* = 25)	12.36 (*N* = 22)	*U* = 139; *p* < 0.01
Percent of time spent in activity (%)			
Mean ± SE	30.83 ± 4.77	6.20 ± 1.97	
Median	45.83 (*N* = 26)	2.00 (*N* = 26)	*U* = 143; *p* < 0.001

**Figure 4:**
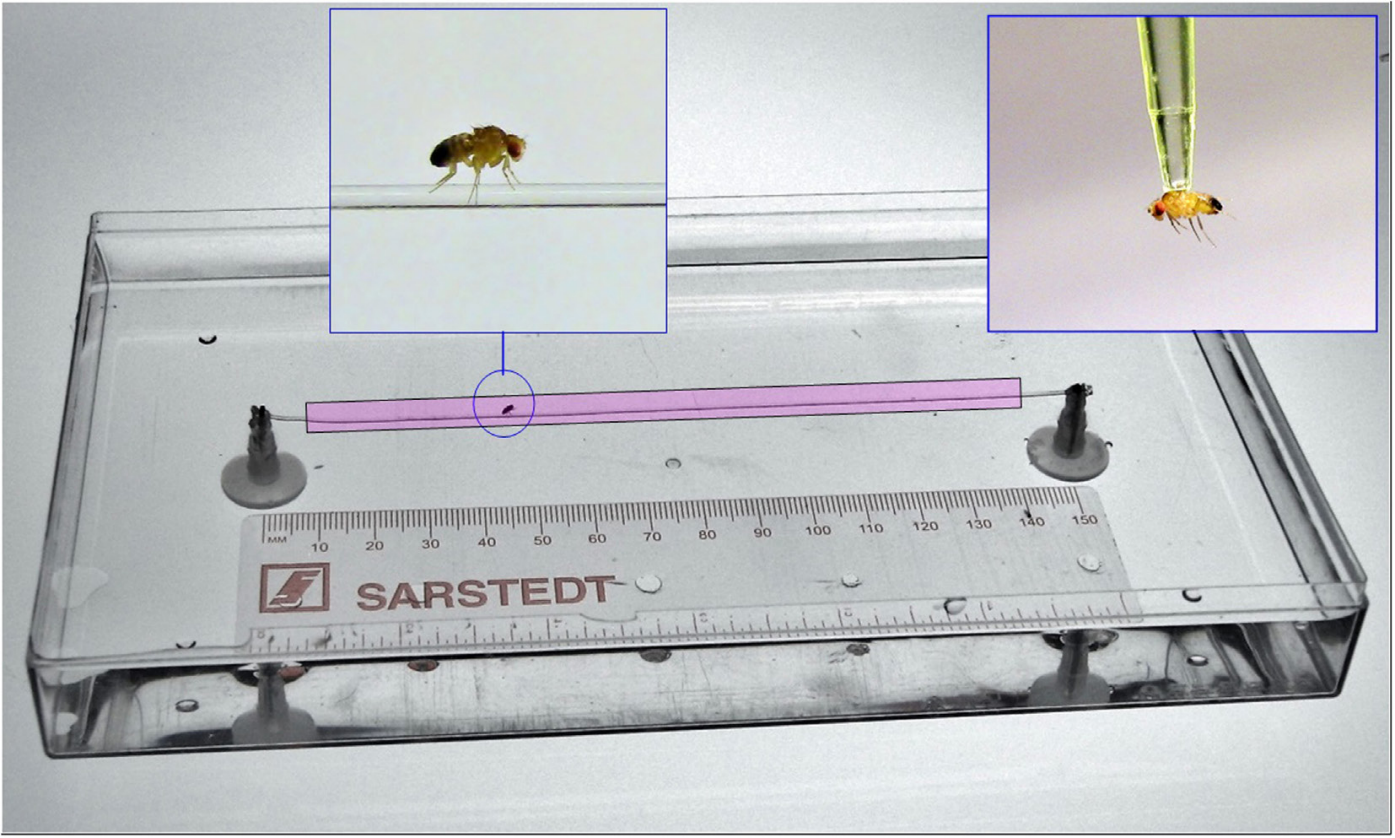
**General view on the experimental setup for measuring *Drosophila* psychomotor performance**. For details, see Section “Materials and Methods.”

Although the open field test allows us to determine if flies exhibit general defects in locomotion, it is not sufficient to reveal impairments in fine-tune movements and/or motor coordination. This is due, in part, to the fact that flies can compensate for small defects in locomotion by the presence of six legs and flexible inter-leg coordination (26). For example, blocking proprioceptive feedback inactivates sensory neurons in the fly’s legs and results in deficient step precision; however, inter-leg coordination and the ability to execute a tripod gait are unaffected (27). In addition, flies show immediate adaptations in body posture, leg kinematics, and inter-leg coordination after removal of a hind leg, thereby, maintaining their ability to walk (26). Therefore, additional methods are required to identify and quantify defects in fine motor movements including balance, gait, coordination, and movement slowing that might be affected in fly models of psychomotor disorders. To this end, we developed a sensitive analytical approach, called the equilibrist test, which measures the ability of a fly to navigate along a fine line suspended between two platforms over a pool of water (Figure 4). To ensure that any differences we observed in the equilibrist test were not simply due to a difference in the size/width of individual flies, we first performed morphometric measurements of control and mutant males. We found no significant differences between control males (0.6215 ± 0.005 mm, *N* = 22) and mutant *IPLA2-VIA* males (0.6237 ± 0.005, *N* = 22; *t*-test, *p* = 0.77).

**Figure 5:**
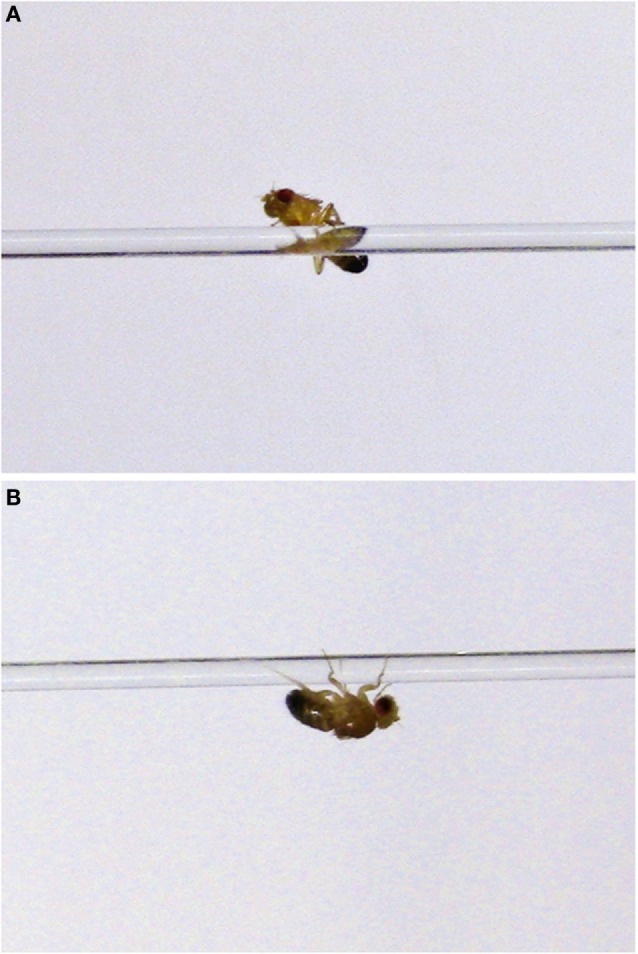
**Examples of balancing on the line (A) or climbing upside down (B) behaviors observed in 4-week-old *IPLA2-VIA* mutant flies during performance in “equilibrist test.”**

To determine whether we could detect the age-related deficits observed in *IPLA2-VIA* mutant flies using open field experiments, we first measured walking speed in 4-week-old flies. However, we were unable to compare the performance between control and *IPLA2-VIA* mutant flies since the majority of mutant males were almost completely inactive. While some mutant flies attempted to move along the line, they spent a lot of time balancing at a specific point on the line, climbing upside down, and eventually falling down into the water (Figure 5). In contrast, 2-week-old flies demonstrated much better performance in this test. Initially, males that were placed on top of the platform jumped into the water irrespective of their genotype. Some flies also attempted to escape from the restricted space (freedom reflex), but since we perform the assay on flies that have had their wings clipped, they can only escape by jumping away or moving along the line. Therefore, after a few attempts, they began to walk along the line linking the two platforms.

**Figure 6:**
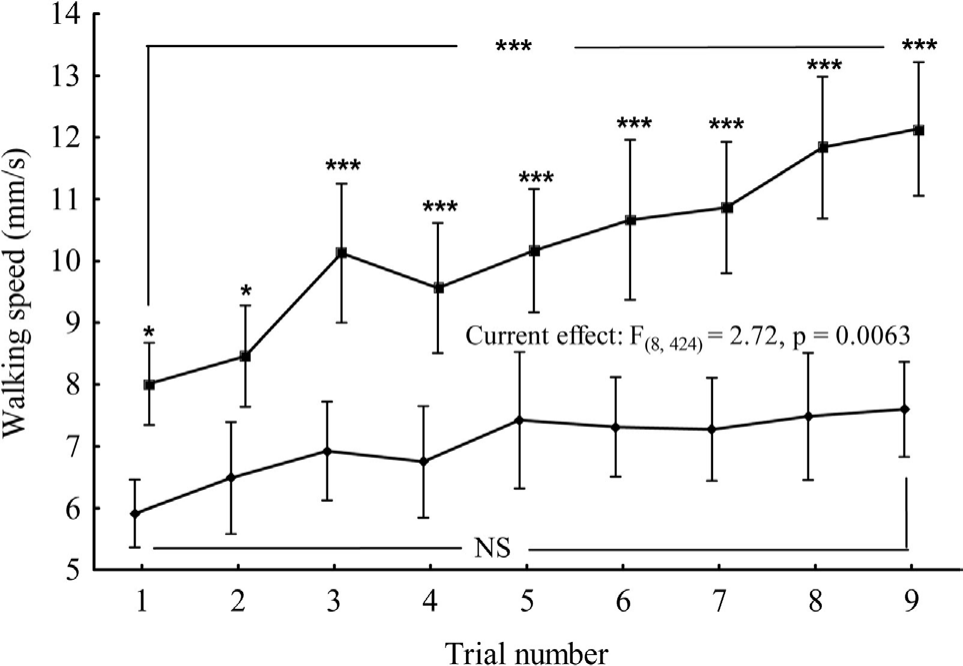
**Mean of the walking speed for control (upper curve) and *IPLA2-VIA* mutant (lower curve) flies plotted as a function of the number of successive trials**. Tukey HSD *post hoc* comparisons show differences between mutant and control in the mean of walking speed for every trial. Vertical bars denote 0.95 confidence intervals. Current effect represents the interaction effect between genotype and number of trials. Also, shown are the comparisons between first and last trial within the same genotype. *denotes significant differences at *p* < 0.05, or ***p < 0.001, NS denotes non-significant.

To determine whether *IPLA2-VIA* mutant males exhibit any differences in walking speed compared with controls and whether the ability of a fly to execute this task can improve from trial to trial, we tested the performance of individual flies during nine successive trials divided into three sessions (1 day apart from each other). For data comparison, we developed a GLM, which is mathematically identical to a multiple regression analysis, but is suited to implement any parametric statistical test with one dependent variable. The walking speed was taken as the dependent variable, whereas genotype and number of trials as categorical predictors. The analysis revealed a significant interaction between genotype and number of trials *F*_(8;424)_ = 2.72; *p* < 0.01 (Figure 6). Further analyses of the walking speed for each genotype revealed a significant positive association between number of trials and walking speed in control flies *F*_(8;243)_ = 7.42; *p* < 0.01 but failed to detect this association in *IPLA2-VIA* mutant flies *F*_(8;234)_ = 1.72; *p* < 0.095. There were also significant differences in walking speed between control and *IPLA2-VIA* mutant flies at the very first trial, as determined by Tukey HSD *post hoc* analysis (control: 8.014 ± 0.32, *N* = 28, mutant: 5.913 ± 0.27, *N* = 27; *p* < 0.014), suggesting that the psychomotor impairments in *IPLA2-VIA* mutant flies appear relatively early and can be detected using the “equilibrist test” whereas they were undetectable using the standard open field test.

## Discussion

Psychomotor is a term that refers to all of the cognitive processes associated with physical movement. It is not just a simple stimulation of motor units through the peripheral motor nerves but rather complex cognitive processes that determine and control whole sequences of actions involved in motor planning, selection, and preparation of motor action and action monitoring (28). Psychomotor learning underlies the development and persistence of patterns of motor activity that are guided by environmental signals. These include motor skills involved in driving, typing, dancing, or athletic performance as well as fine skills used to control precision instruments and tools. Interestingly, disturbances in psychomotor activity are an important feature of many neuropsychiatric and mood disorders (9–11, 29).

Interestingly, similar to other human neurodegenerative disorders, *PLA2G6*-associated neurodegeneration also seem to be age-dependent. Early onset (infants) of INAD is characterized by progressive psychomotor and mental retardation. Many affected children unable learn to walk or very quick loss this ability. In the atypical form of this disease (early childhood), the most common signs are gait instability or ataxia, psychomotor regression, and autistic-like behavior. Late onset of these disorders (adulthood) is typically associated with several predominant features as a Parkinsonism (tremor, rigidity, and markedly impaired postural responses) and cognitive decline.

Here, we show that *iPLA2-VIA* mutant flies, the *Drosophila* homolog of the human *PLA2G6*, develop age-dependent psychomotor impairments. One of the striking findings of the present study is that wild type flies were able to improve their motor skills by repeated performance. According to classical models, when acquiring psychomotor skills, a learner makes transitions through cognitive, associative, and autonomous stages (30). At the first stage, the learner has to think before start doing the movement. The performance at this stage is generally slow and contains many errors. In the next stage, the learner associates the movement that is already known with one they are learning and spend less time thinking about the details. In the final stage, the learning is almost done and movements become autonomous. What kind of memory is most directly involved in this phenomenon is not clear yet. However, it is generally accepted that psychomotor behavior is best remembered (and least forgotten) when overlearning is high, reinforcing feedback is optimal, and interpolated activities are unrelated to the task being learned (31). Whether similar rules govern psychomotor learning in flies has yet to be determined.

In summary, we have developed a novel assay that can be used to measure fine-tune motor movements, motor coordination, and psychomotor learning in wild type *Drosophila*.

We then used this assay to measure psychomotor behavior in wild type and flies carrying a mutation in *IPLA2-VIA*, which is associated with progressive neurodegeneration and psychomotor deficits in humans. We found that an equilibrist assay is sensitive enough to detect defects in fine-tune movements or motor coordination in the *IPLA2-VIA* mutants at younger ages (2 weeks) that were not detected using the open field assay. We also found that mutant flies exhibit defect in psychomotor learning.

## Author Contributions

KI designed the experiments, acquired, analyzed the data, and wrote the manuscript. OG acquired data for the manuscript. GB analyzed the data, revised the manuscript, and is accountable for all aspects of the work.

## Conflict of Interest Statement

The authors declare that the research was conducted in the absence of any commercial or financial relationships that could be construed as a potential conflict of interest.
